# Antiviral Innate Immune Response Interferes with the Formation of Replication-Associated Membrane Structures Induced by a Positive-Strand RNA Virus

**DOI:** 10.1128/mBio.01991-16

**Published:** 2016-12-06

**Authors:** Diede Oudshoorn, Barbara van der Hoeven, Ronald W. A. L. Limpens, Corrine Beugeling, Eric J. Snijder, Montserrat Bárcena, Marjolein Kikkert

**Affiliations:** a Molecular Virology Laboratory, Department of Medical Microbiology, Leiden University Medical Center, Leiden, The Netherlands; b Section Electron Microscopy, Department of Molecular Cell Biology, Leiden University Medical Center, Leiden, The Netherlands

## Abstract

Infection with nidoviruses like corona- and arteriviruses induces a reticulovesicular network of interconnected endoplasmic reticulum (ER)-derived double-membrane vesicles (DMVs) and other membrane structures. This network is thought to accommodate the viral replication machinery and protect it from innate immune detection. We hypothesized that the innate immune response has tools to counteract the formation of these virus-induced replication organelles in order to inhibit virus replication. Here we have investigated the effect of type I interferon (IFN) treatment on the formation of arterivirus-induced membrane structures. Our approach involved ectopic expression of arterivirus nonstructural proteins nsp2 and nsp3, which induce DMV formation in the absence of other viral triggers of the interferon response, such as replicating viral RNA. Thus, this setup can be used to identify immune effectors that specifically target the (formation of) virus-induced membrane structures. Using large-scale electron microscopy mosaic maps, we found that IFN-β treatment significantly reduced the formation of the membrane structures. Strikingly, we also observed abundant stretches of double-membrane sheets (a proposed intermediate of DMV formation) in IFN-β-treated samples, suggesting the disruption of DMV biogenesis. Three interferon-stimulated gene products, two of which have been reported to target the hepatitis C virus replication structures, were tested for their possible involvement, but none of them affected membrane structure formation. Our study reveals the existence of a previously unknown innate immune mechanism that antagonizes the viral hijacking of host membranes. It also provides a solid basis for further research into the poorly understood interactions between the innate immune system and virus-induced replication structures.

## IMPORTANCE

Viruses with a positive-strand RNA genome establish a membrane-associated replication organelle by hijacking and remodeling intracellular host membranes, a process deemed essential for their efficient replication. It is unknown whether the cellular innate immune system can detect and/or inhibit the formation of these membrane structures, which could be an effective mechanism to delay viral RNA replication. In this study, using an expression system that closely mimics the formation of arterivirus replication structures, we show for the first time that IFN-β treatment clearly reduces the amount of induced membrane structures. Moreover, drastic morphological changes were observed among the remaining structures, suggesting that their biogenesis was impaired. Follow-up experiments suggested that host cells contain a hitherto unknown innate antiviral mechanism, which targets this common feature of positive-strand RNA virus replication. Our study provides a strong basis for further research into the interaction of the innate immune system with membranous viral replication organelles.

## INTRODUCTION

All positive-strand RNA viruses of eukaryotes studied to date modify intracellular membranes into unique structures that presumably facilitate viral RNA synthesis. These can therefore be viewed as the “headquarters” of positive-strand RNA viral replication (1–4). Elaborate interactions between virus and host are believed to form the basis for the striking, virus-induced remodeling of specific cellular organelles in the infected cell (5–8). These replication organelles may consist of different substructures, such as spherules, tubules, convoluted membranes, paired membranes, or double-membrane vesicles. Despite this diversity, two recurrent classes of replication organelles induced by positive-strand RNA viruses have been recognized. The first type consists of membrane invaginations that create small “spherules” in the membranes of intracellular organelles or the plasma membrane. Neck-like connections between the cytosol and the interior of the spherule, in which RNA synthesis takes place, are presumed to facilitate transport of viral RNA products to the cytosol for translation and packaging. Spherules of this kind have been described for, e.g., alphaviruses, some flaviviruses, nodaviruses, and bromoviruses (9–12). The second type of structure is characterized by unique membrane tubules and/or vesicles that have a double membrane. During the past decade, this kind of membrane structure has been observed and characterized extensively by electron tomography for arteriviruses, coronaviruses, picornaviruses, and hepatitis C virus (HCV) (13–18). For some of these double-membrane vesicle (DMV)-forming viruses, connections between the DMV interior and the cytosol have been observed (14–16). However, this was not the case for arteri- and coronaviruses, raising the question of whether their RNA synthesis takes place inside these vesicles, by analogy with the replication spherules described above. In that scenario, it would be unclear how newly synthesized RNA molecules are exported to the cytosol for translation and packaging (13, 17).

Equine arteritis virus (EAV) is the prototype of the arterivirus family and induces the formation of an extensive reticulovesicular network (RVN) of DMVs, which is thought to be derived from the endoplasmic reticulum (ER) (13). Furthermore, EAV infection results in the formation of proteinaceous tubules containing the nucleocapsid (N) protein, which were found in close proximity to the DMVs and were suggested to be involved in nucleocapsid assembly (13, 19). The EAV replicase gene encodes two large polyproteins, pp1a and pp1ab, the second being a C-terminally-extended version of the first produced by ribosomal frameshifting. These precursors are cleaved by internal proteases to yield at least 13 mature viral nonstructural proteins (nsps) (reviewed in reference 20). Previous studies from our laboratory provided the first detailed description of arterivirus-induced remodeling of host membranes and established that expression of the nsp2 to -7 part of the EAV replicase polyproteins suffices to induce the formation of DMVs strikingly similar to those formed in infected cells (21). Subsequent studies demonstrated that the same result can be achieved by expressing a polyprotein fragment encompassing nsp2 and nsp3, including the papain-like protease in nsp2 (PLP2) that cleaves the nsp2-nsp3 junction (22, 23). As for other positive-strand RNA viruses, the viral nonstructural proteins directly involved in RNA synthesis, such as the RNA-dependent RNA polymerase (RdRp), as well as the (presumed) double-stranded RNA (dsRNA) intermediates of viral RNA replication, colocalize with the membrane structures induced during EAV infection (1, 13, 21). When analyzed in more detail, however, the dsRNA was mainly located in the core of the DMVs, whereas the nonstructural proteins were located mainly on the membranes of the vesicles as well as on surrounding membranes (13, 21), as was also observed for coronaviruses (17, 24).

How replication organelles are formed during infection is still largely unclear. One proposed mechanism for DMV biogenesis, termed “double budding,” includes the acquisition of the double membrane by the sequential budding of vesicles into and out of the ER lumen. Alternatively, ER membranes may pair to form double-membrane sheets, which would then bend and undergo fission to produce closed vesicles, a process termed “enwrapping” (1, 21, 23). In a recent study, we detected intermediate structures compatible with both models. Putative intermediates consisting of double-membrane sheets that fitted different stages of enwrapping were, however, particularly prominent, suggesting that enwrapping could be a key biogenesis pathway for EAV-induced structures (23). In the present study, we will refer to any combination of EAV nsp-induced membrane structures, including these intermediates, as “double-membrane structures” (DMS), irrespective of whether they are formed in the context of viral infection or upon expression of viral proteins.

The exact benefits of positive-strand RNA viral replication organelle formation remain unclear, although several advantages have been proposed (1–3, 23, 25). First, the replication organelles could constitute a suitable microenvironment for viral RNA synthesis by concentrating the necessary viral and host proteins. Second, they could play an important role in the spatiotemporal coordination of the different steps of the viral replication cycle, such as genome translation, replicase polyprotein processing, RNA synthesis, and virion assembly. The third proposed role is hiding viral RNA species from detection by innate immune sensors. These sensors recognize pathogen-associated molecular patterns (PAMPs) such as virus-derived nucleic acids, leading to the induction of inflammatory responses and the production of type I interferons such as beta interferon (IFN-β) (26–28). These interferons then signal in autocrine and paracrine fashion to upregulate the expression of interferon-stimulated genes (ISGs) inducing a so-called “antiviral state” that strongly restricts further spread of the infection (26–28). The antiviral activities of several of the hundreds of ISGs induced upon type I interferon signaling have been characterized to a certain extent, and these impact diverse aspects of viral infection such as entry, genome replication, particle formation, or budding (29). Even though virus-induced replication organelles are a prominent feature of positive-strand RNA viruses, there is very limited evidence for a direct targeting of these replication organelles by the innate immune system. To our knowledge, in fact only two reports have described such effects—in both cases on the membranous web formed during HCV replication (30, 31); however, the underlying mechanisms remain to be fully characterized, and data for any other positive-strand RNA virus are lacking.

We here studied the effects of type I IFN-induced signaling on DMS formation induced by EAV nsp expression, measured using quantitative electron microscopy (EM) methods that allowed the direct evaluation of the impact of the innate immune response on the formation of EAV-induced DMSs. We observed that IFN-β treatment reduces the number of cell sections showing DMSs and drastically changed the morphology of the remaining structures. In order to investigate the underlying mechanism, we evaluated the role of individual ISGs, including cholesterol 25-hydroxylase (CH25H) and viperin, which are known to inhibit HCV membranous web formation (30, 31). However, the candidates tested appear not to be involved in restricting EAV nsp-induced membrane remodeling. This suggests the existence of a previously unknown IFN-β-induced mechanism targeting the formation of the replication organelles induced by arteriviruses and—possibly—other positive-strand RNA viruses.

## RESULTS

### Experimental setup for studying the interaction of the innate immune system with arterivirus-induced membrane structures.

**FIG 1:**
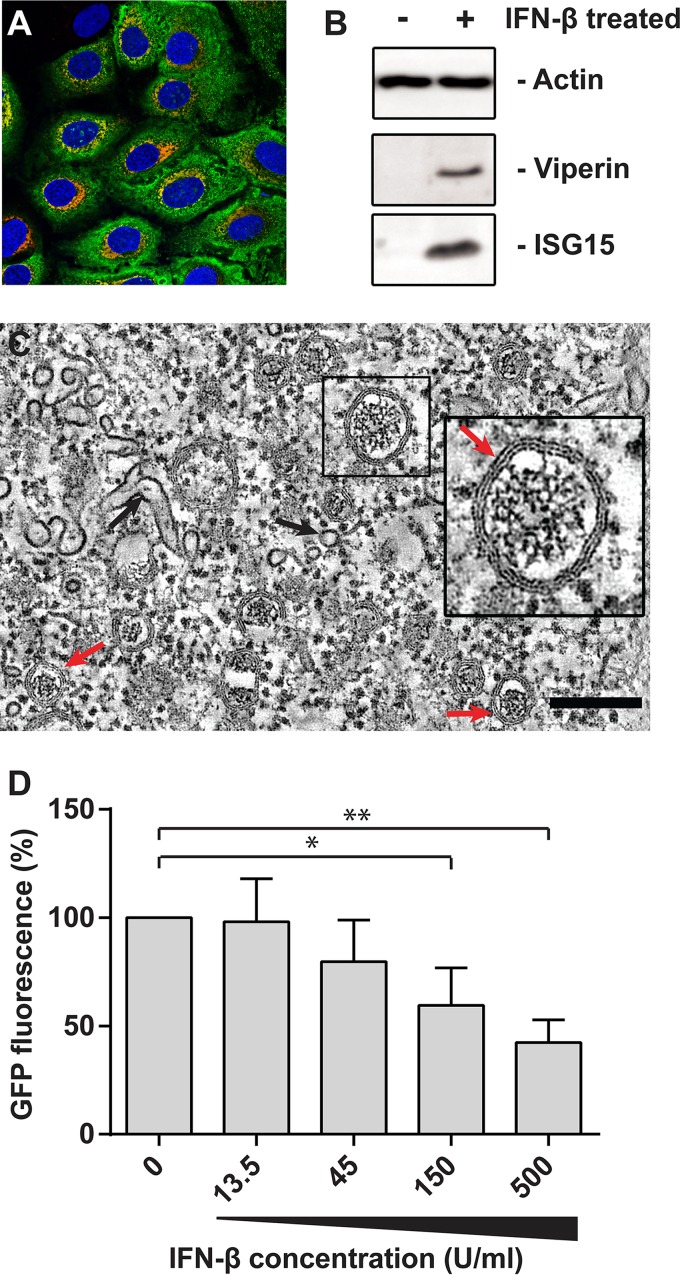
EAV infection in HuH-7 cells is productive and sensitive to IFN-β treatment. (A) HuH-7 cells infected with EAV were fixed and stained with antibodies specific for nsp2 (red) and nucleocapsid protein (N; green) at 12 hpi. The nuclei were stained with Hoechst 33258 (blue). (B) Protein levels in HuH-7 cells were analyzed by SDS-PAGE followed by Western blotting after 12 h of stimulation with 500 U/ml IFN-β or were analyzed in untreated control cells. (C) EAV-infected HuH-7 cells were high-pressure frozen at 12 hpi. Samples were freeze substituted and analyzed by electron tomography as previously described (23). A virtual slice (2 nm thick) of a reconstructed tomogram is shown. The red arrows point at some of the multiple DMVs present in the field of view. The boxed DMV is enlarged in the inset to make the two tightly apposed DMV membranes more apparent. Black arrows indicate some of the N-containing tubules. The scale bar represents 200 nm. (D) HuH-7 cells were infected with EAV-GFP in 96-well black plates for 1 h, and after removal of the inoculum, they were treated with the indicated dose of IFN-β. Cells were fixed at 16 hpi, and GFP fluorescence was measured and normalized to a control infection. Error bars represent the standard deviation from 3 independent experiments, and statistical analysis was done with an unpaired Student’s *t* test. *, *P* < 0.05; **, *P* < 0.01.

Our goal was to analyze whether the innate immune system responds to the formation of positive-strand RNA virus-induced replication organelles, and we hypothesized that such a response could be linked to the type I IFN signaling pathway, which has an important role in counteracting virus infections from their earliest stage onwards. Human liver carcinoma cells (HuH-7), which are susceptible to EAV infection (Fig. 1A) (23) and produce high titers of infectious virus, were used based on their responsiveness to IFN-β treatment (Fig. 1B) (32). When EAV-infected HuH-7 cells were fixed by high-pressure freezing followed by freeze substitution (HPF-FS) and subsequently analyzed by EM, DMSs similar to those previously described upon EAV infection in other cell types were readily observed (Fig. 1C) (13, 23). DMVs with characteristic double membranes and cores were abundantly present (Fig. 1C, red arrows) as well as the N protein-containing tubules described previously (13) (Fig. 1C, black arrows). In order to establish whether EAV replication is sensitive to IFN-β treatment in HuH-7 cells, cells were infected with a recombinant green fluorescent protein (GFP)-expressing reporter virus (EAV-GFP [33]; multiplicity of infection [MOI] of 10) and treated with IFN-β from 1 h postinfection (p.i.) onward. A clear dose-dependent reduction of the GFP signal was observed during a single cycle of infection (Fig. 1D).

Because DMS formation requires the accumulation of viral nsps (21) derived from the abundant replication and translation of the viral RNA genome, it is not possible to study the effect of IFN-β treatment on DMS formation directly in infected cells, since the treatment will inhibit overall viral replication and—consequently—nsp synthesis. Thus, to investigate innate immune responses specifically targeting DMS formation, the latter process needed to be mimicked in a system that does not depend on EAV replication. Previously, the coexpression of EAV nsp2 and nsp3 (as a self-cleaving nsp2-3 polyprotein fragment) has been shown to be both required and sufficient to induce DMV formation in transfected cells, and such a system can thus be used as a “surrogate” to mimic the formation of DMSs outside the context of infection (22).

**FIG 2:**
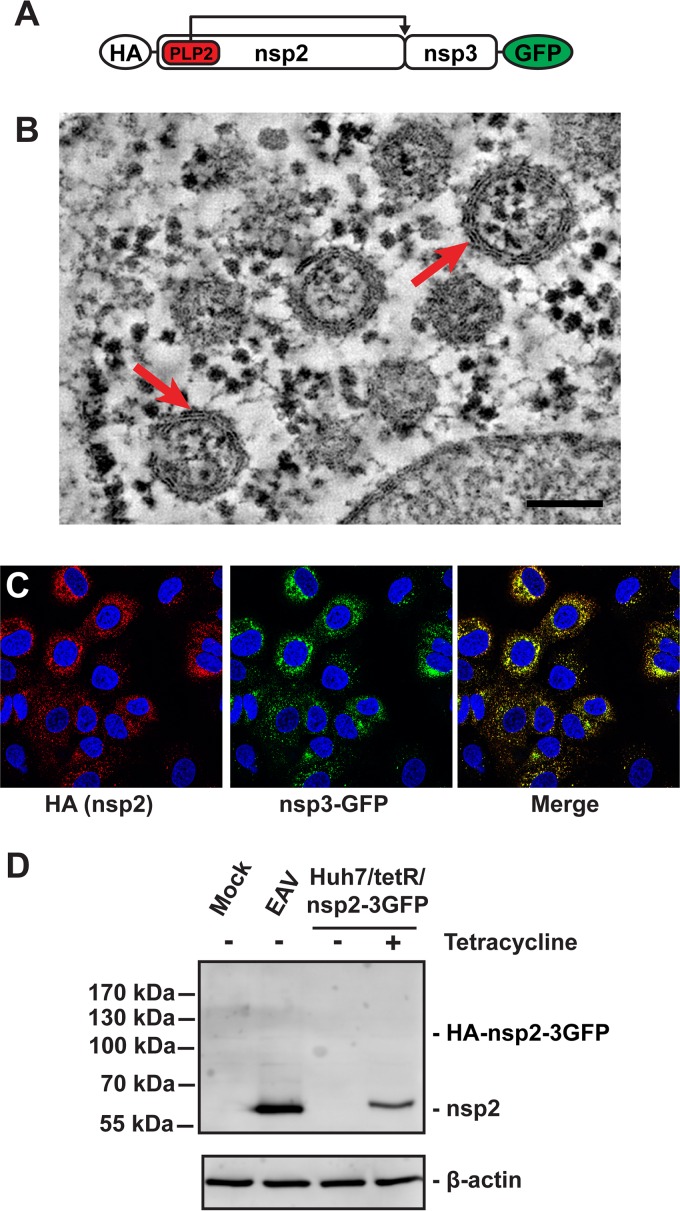
Validation of the HuH-7/tetR/HA-nsp2-3GFP cell line. (A) Schematic overview of the HA-nsp2-3GFP protein. (B) HuH-7/tetR/HA-nsp2-3GFP cells were high-pressure frozen, followed by freeze substitution and subsequently analyzed by electron tomography as described before (23). A 2-nm-thick virtual slice of a reconstructed tomogram is shown. The red arrows indicate some of the DMVs that can be clearly observed. The scale bar represents 100 nm. (C) Immunofluorescence microscopy of HuH-7/tetR/HA-nsp2-3GFP cells treated with tetracycline for 24 h to induce expression. Cells were stained with mouse anti-HA and the HA-nsp2 signal was detected using a Cy3-conjugated antibody, whereas as nsp3GFP could be visualized by virtue of its fluorescent tag. (D) EAV nsp2 expression was analyzed by Western blotting during infection (12 hpi) and in induced HuH-7/tetR/HA-nsp2-3GFP cells (24 h after induction).

In order to develop a stable and inducible expression system, we generated polyclonal HuH-7 cell lines expressing EAV nsp2 and -3 with a hemagglutinin (HA) tag at the nsp2 N terminus and a C-terminal GFP tag on nps3 under the control of a tetracycline-inducible cytomegalovirus (CMV) promoter (HuH-7/tetR/HA-nsp2-3GFP [Fig. 2A]) (34). Induction of nsp2-3 expression in this cell line resulted in the formation of uniform DMVs (Fig. 2B), which appeared similar to those observed in HuH-7-infected cells, whereas such structures were not observed in noninduced cells. Although these DMVs were slightly larger than those found during EAV infection and (as expected) lacked the RNA-containing electron-dense core (compare Fig 1C and 2B), electron tomography showed that their double-membrane architecture was identical (Fig. 2B). At 24 h post-tetracycline induction, many DMVs were found (Fig. 2B). The presence of the C-terminal GFP tag on nsp3 did not influence the morphology of DMVs, as they were indistinguishable from those induced by expression of HA-nsp2-3 lacking the GFP tag (23). The nsp2-3 expressed in HuH-7/tetR/HA-nsp2-3GFP cells localized to the perinuclear region (Fig. 2C), which is very similar to their localization in EAV-infected cells (Fig. 1A). While the expression levels of nsp2 were somewhat lower in the tetracycline-induced HuH-7/tetR/HA-nsp2-3GFP cells compared to that in EAV-infected cells, the nsp2-3 polyprotein was correctly and efficiently cleaved by PLP2, as no precursor protein could be observed (Fig. 2D). In conclusion, this cell line could be used to reproducibly and quantitatively examine the specific interactions of arterivirus-induced membrane structures with the cell’s innate immune responses.

### IFN-β treatment disrupts the formation of double-membrane structures.

**FIG 3:**
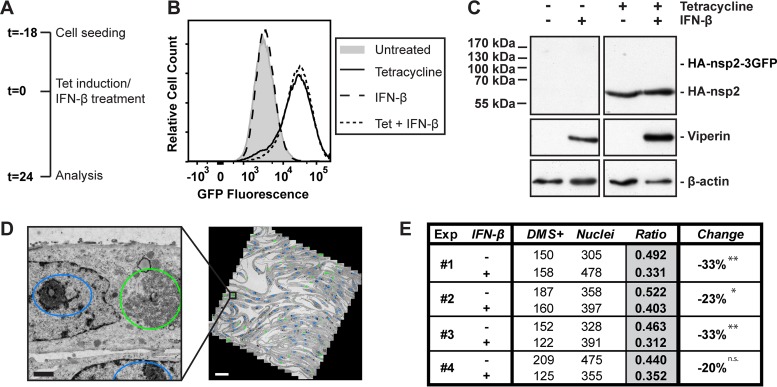
IFN-β treatment reduces the number of double-membrane structures formed by EAV nsp2-3, while protein levels and nsp2-3 cleavage efficiency are not affected. (A) Schematic overview of the experimental setup. (B) HuH-7/tetR/HA-nsp2-3GFP cells were analyzed by flow cytometry for GFP fluorescence after 24 h of the indicated treatments. (C) Levels of the indicated proteins in HuH-7/tetR/HA-nsp2-3GFP cells were analyzed 24 h after the indicated treatments, using Western blotting. The expected position of the HA-nsp2-3GFP precursor is indicated. (D) Example of a mosaic map (right) of a single mesh of an EM grid (tetracycline-treated HuH-7/tetR/HA-nsp2-3GFP cells) composed of 1,164 images (2,048 by 2,048 pixels each) acquired at 6,800× magnification and binning 2, which corresponded to a pixel size of 3.2 nm. The closeup (left) was extracted from the mosaic map as indicated. Coloring represents annotations of nuclei (blue ovals) and EAV nsp2-3-induced DMS (green ovals) in this mesh. Scale bars represent 500 nm (left) and 20 µm (right), respectively. (E) In four independent experiments, the number of cell profiles positive for DMS (DMS^+^) was quantified as well as the number of cell profiles containing a nucleus (Nuclei). Multiple mosaic maps were analyzed for each sample. Ratios are calculated as the number of DMS^+^ cell profiles divided by the number of cell profiles containing a nucleus, and *P* values were calculated using chi-square tests for each experiment. *, *P* < 0.05; **, *P* < 0.01. n.s., not significant. The average reduction over 4 experiments was 27% ± 7% (*P* = 0.001).

We first checked whether the expression of nsp2-3GFP in HuH-7/tetR/HA-nsp2-3GFP cells and the resulting DMV formation by itself induced an innate immune response, which would imply that the structures can be sensed by the innate immune system. In our setup, this was not the case since no IFN-β or IFIT2 mRNA could be detected after induction of nsp2-3GFP expression in HuH7 or mouse embryonic fibroblast (MEF) cells (data not shown). We argued that in EAV-infected cells type I IFN induction might be triggered by other viral PAMPs (e.g., viral RNA), which could affect the formation or function of the replication organelles by inducing the expression of particular ISGs and thus augmenting the general antiviral effect of the innate immune response. In order to address this possibility, we initially asked the question of whether IFN-β treatment affects the number of EAV nsp2-3-induced DMS formed per cell. For this time-consuming quantitative analysis, we decided to use chemically fixed samples, which simplified the workflow while providing sufficient preservation to clearly assess the general morphology of the DMSs induced upon nsp2-3 expression (see below). The HuH-7/tetR/HA-nsp2-3GFP cells were chemically fixed, scraped from the dish, pelleted, and embedded in an epoxy resin, after which thin sections suitable for transmission EM imaging were cut. By using cell pellets rather than monolayers, these sections represented random planes through the cells. We argued that if there would be a difference in the extent of DMS formation, this would be reflected in the number of cell profiles that contain DMSs, as the fraction of random sections containing structures would decrease. Importantly, we first checked the effect of IFN-β treatment on nsp2-3 expression levels and polyprotein cleavage in HuH-7/tetR/HA-nsp2-3GFP cells, since altered expression of nsp2-3 would likely affect DMS formation and possibly mask specific effects of the IFN-β treatment. Cells were treated with tetracycline and/or IFN-β (Fig. 3A), and nsp3GFP expression was measured by flow cytometry. GFP signal increased after tetracycline induction, as expected, but was not affected by IFN-β treatment at the concentration used in our experiments (Fig. 3B). Likewise, polyprotein cleavage was not majorly affected since no HA-nsp2-3GFP precursor could be observed after IFN-β treatment, similar to the situation without IFN-β treatment (Fig. 3C).

After setting up this system for DMS quantification, the next step was to quantify nsp2-3-induced DMS in HuH-7/tetR/HA-nsp2-3GFP cells, treated with tetracycline alone or in combination with IFN-β. Using large mosaic maps of EM micrographs (35), we quantified the number of cell profiles positive for DMSs as well as the total number of cell profiles showing a nucleus, which was used as a reference for normalizing the total number of cells analyzed (Fig. 3D). In four independent experiments, sections containing 300 to 500 cell profiles with a nucleus were analyzed for each condition. This revealed a consistent decrease (27% ± 7%) in the number of cell profiles positive for nsp2-3-induced DMSs after treatment with IFN-β (Fig. 3E). Because the nsp2-3 expression levels were very similar between the samples (Fig. 3B), our conclusion is that the induction of DMV formation by EAV nsp2 and nsp3 was substantially inhibited and that the type I interferon treatment restricts the hijacking of cellular membranes.

**FIG 4:**
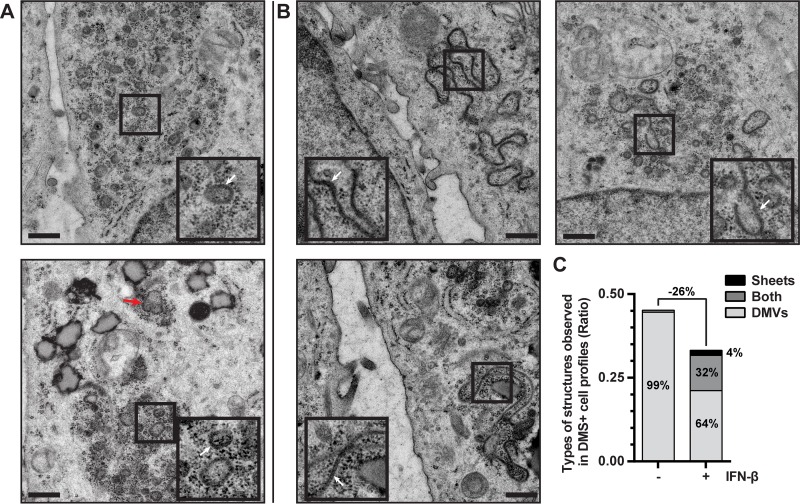
IFN-β treatment blocks DMV formation and leads to the accumulation of double-membrane sheets. (A and B) EM images of HuH-7/tetR/HA-nsp2-3GFP cells in which expression is induced with tetracycline for 24 h. Cells represented in images of panel B were simultaneously treated with 500 U/ml IFN-β for 24 h. Images were extracted from mosaic maps used for quantifications, and scale bars represent 500 nm. Insets are 2× magnifications of areas where DMVs (A) or double-membrane sheets (B) are visible (indicated with white arrows). The different appearance of these samples relative to the data shown in Fig. 2 is the result of the different sample preparation approaches (chemical fixation versus HPF-FS, respectively). (C) The occurrence of the different types of DMS (DMVs, double-membrane sheets, or both) in the cell profiles is shown in control or IFN-β-treated HuH-7/tetR/HA-nsp2-3GFP cells that were tetracycline induced.

Besides the changes in their abundance caused by IFN-β treatment, we also analyzed the morphology of the DMS. As mentioned previously, tetracycline-induced HuH-7/tetR/HA-nsp2-3GFP cells mainly developed the DMV type of DMS (Fig. 4A). In 1% of the DMS-containing cell profiles, we also observed some double-membrane sheets, a presumed precursor of DMVs during their biogenesis (Fig. 4A, bottom panel, red arrow) (21, 23). These sheets were mostly found in the vicinity of DMVs and resembled the two tightly apposed membranes of the DMVs themselves, but they varied in shape, depending in part on the sectioning plane for EM. When the tetracycline-induced HuH-7/tetR/HA-nsp2-3GFP cells were also treated with IFN-β, double-membrane sheets were found in much larger fraction of the DMS-containing cell profiles (36% ± 3%) (Fig. 4B and C). With a few exceptions, the double-membrane sheets were found in cell profiles that also contained DMVs (Fig. 4B and C). They were often juxtaposed to intact DMVs and were usually strikingly more extensive than the double-membrane sheets observed without IFN-β treatment (compare Fig. 4A [bottom panel, red arrow] and B). This strong increase in double-membrane sheet formation suggested a major effect in DMS biogenesis after IFN-β treatment.

### Involvement of ISGs in inhibition of DMV formation.

Likely, the effects of IFN-β treatment on DMS morphology and abundance are caused by the products of one or more ISGs, which are expressed as a result of the IFN-β treatment. Since EM is required to distinguish DMVs from double-membrane sheets, a high-throughput screen of the hundreds of ISG candidates was not feasible. Interestingly, two membrane-associated ISGs, viperin (also called radical *S*-adenosyl methionine domain-containing protein 2 [RSAD2]) and CH25H (the latter resulting in production of effector 25-hydroxy cholesterol [25HC]) were previously shown to influence HCV replication membranes, which have a similar double-membrane architecture to that described for arteriviruses (25, 30, 31). Anggakusuma and coworkers expressed HCV NS3-5B proteins in HuH-7 cells, which resulted in the formation of membrane structures similar to those found upon HCV infection (15). Additional treatment of these cells with 25HC triggered, among other changes, the formation of smaller DMVs, leading to the conclusion that 25HC influences the HCV-induced membrane structures (30). The changed DMVs these authors observed were different from the double-membrane sheets that accumulate in our EAV nsp2-3 expression system upon IFN-β treatment.

**FIG 5:**
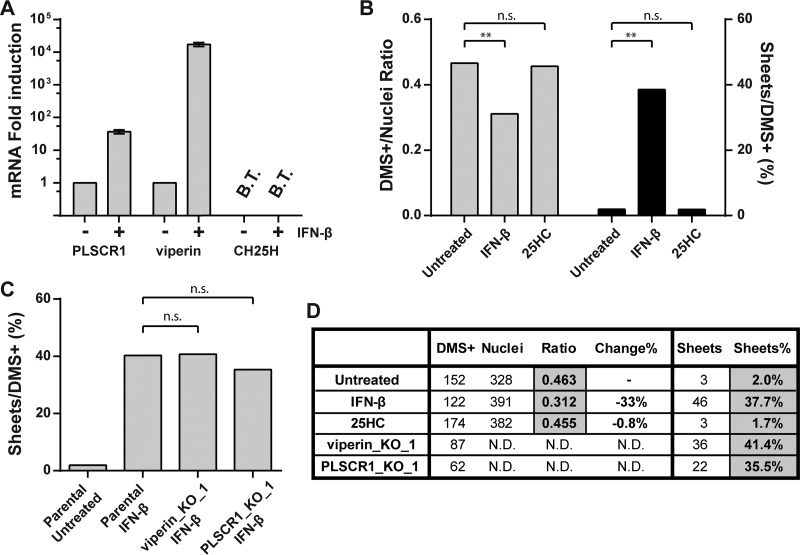
CH25H, PLSCR1, and viperin are not involved in the inhibition of EAV nsp2-3 DMV formation by IFN-β. (A) Relative induction of indicated ISG mRNAs in HuH-7 cells treated with IFN-β for 16 h compared to untreated control cells, using reverse transcriptase quantitative PCR (RT-qPCR) analysis. Indicated genes were amplified with gene-specific primers, error bars were based on three independent experiments. CH25H mRNA was below the threshold of detection (B.T.) in HuH-7 cells. The CH25H RT-qPCR was validated using cDNA of lipopolysaccharide-treated human dendritic cells. (B) Tetracycline-induced HuH-7/tetR/HA-nsp2-3GFP cells were treated with 500 U/ml IFN-β or 10 µM 25HC or untreated and analyzed by EM for the total number of cells positive for DMS (DMS^+^) as well as cell profiles positive for a nucleus (Nuclei). A section covering 300 to 500 cell profiles with a nucleus was analyzed for each sample. Ratios are calculated as the number of DMS^+^ cell profiles divided by the number of nucleus-positive cell profiles as well as the percentage of difference from the untreated control. The number of cell profiles positive for double-membrane sheets was also quantified, and the ratio of sheet-positive cell profiles compared to the total number DMS^+^ cell profiles was determined. (C) Tetracycline-induced CRISPR/Cas9 knockout cell lines of indicated genes were treated with 500 U/ml IFN-β and compared to the parental control cells. The analysis was similar to that for panel B, except that the DMS^+^/nucleus ratio was not determined. (D) A table of the quantifications shown in panels B and C. Statistical analyses were done using chi-square tests. **, *P* < 0.01. n.s., not significant; ND, not determined.

In order to evaluate whether either of these ISGs may play a role in the biogenesis of EAV membrane structures, we first examined the expression of viperin, CH25H, and a third membrane-associated ISG, phospholipid scramblase 1 (PLSCR1), in parental HuH-7 cells upon IFN-β treatment. The mRNAs of both viperin and PLSCR1 were strongly upregulated, whereas CH25H mRNA could not be detected, either with or without IFN-β treatment (Fig. 5A). Although the observation that CH25H was not expressed in HuH-7 cells after IFN-β treatment indicated that this factor could not be responsible for the observed effects in our experiments, we decided to test its impact on EAV nsp2-3-induced DMS, since this IFN-induced factor could be relevant during a natural infection if produced by immune cells. We treated HuH-7/tetR/HA-nsp2-3GFP cells with 10 µM 25HC, a concentration that partially inhibited EAV replication in parental HuH-7 cells (see Fig. S1 in the supplemental material). We, however, found no significant reduction in the number of DMS-containing cells, nor did we observe an increase of double-membrane sheet formation as observed after IFN-β treatment (Fig. 5B to D). We therefore ruled out 25HC synthesis by CH25H as a mechanism that could be responsible for the effects we observed of IFN-β treatment on EAV nsp2-3-induced DMS.

To determine the impact of viperin and PLSCR1 on EAV nsp2-3GFP-induced DMS, we used CRISPR/Cas9 technology to knock out either PLSCR1 or viperin expression in the HuH-7/tetR/HA-nsp2-3GFP cell line, which indeed abolished their expression upon IFN-β treatment (see Fig. S2 in the supplemental material). If either viperin or PLSCR1 was required for the effects of IFN-β treatment we observed (Fig. 4), IFN-β treatment should no longer lead to the proliferation of double-membrane sheets in these respective knockout cells. When the DMSs in tetracycline-induced and IFN-β-treated knockout cell lines were examined, we found no significant differences in the fraction of cells showing double-membrane sheets compared to the parental HuH-7/tetR/HA-nsp2-3GFP cell line (Fig. 5C and D). Together, these data suggest that viperin and PLSCR1 do not contribute to the disruption of nsp2-3-induced DMV biogenesis.

Strikingly, our results imply that, in the same cell line, HCV- and EAV-induced membrane structures, which both include DMVs, are targeted by different IFN-induced effectors. For HCV, 25HC treatment led to the formation of smaller DMVs, whereas for EAV, DMV biogenesis was not affected by 25HC. Our study thus suggests the existence of an alternative innate immune mechanism that antagonizes the viral hijacking of host membranes.

## DISCUSSION

**FIG 6:**
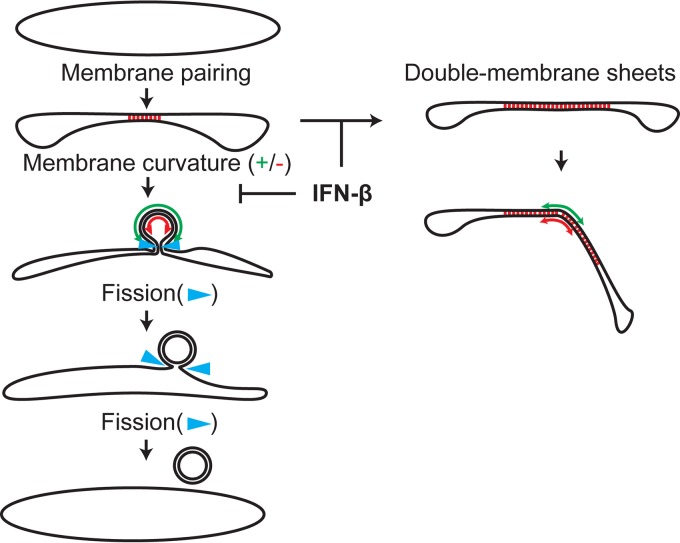
Model for the inhibition of DMV formation by IFN-β treatment. The enwrapping model for DMV formation is shown. Membrane pairing is indicated with red dashes between the membranes. Positive and negative membrane curvatures (green and red, respectively) are indicated with double arrows. The step proposed to be inhibited by IFN-β treatment and resulting in the formation of double-membrane sheets is indicated. Adapted from van der Hoeven et al. (23).

Modification of host membranes to accommodate the viral RNA replication machinery appears to be an essential and universal feature of positive-strand RNA virus infection. Our hypothesis was that—given their abundance in the cytosol of the infected cell—these structures are a likely target of the innate immune system. Disrupting the formation and/or function of virus-induced replication organelles would hamper viral replication and could therefore constitute an effective antiviral strategy for the cell. Our findings show that treatment of cells with IFN-β (mimicking the triggering of innate immunity after recognition of PAMPs such as viral nucleic acids) inhibits the formation of arterivirus-induced DMVs. We not only observed a decrease in the fraction of cells positive for EAV nsp2-3-induced DMS, but we also noticed the extensive accumulation of double-membrane sheets, which could be intermediates of DMV morphogenesis that become much more prevalent upon IFN-β treatment. This suggests that the membrane-curving and/or fission events that could lead to DMV formation after initial membrane pairing (23) were inhibited by IFN-β treatment (Fig. 6). This conclusion is further supported by a preliminary experiment (data not shown) in which expression of nsp2-3GFP was first induced for 24 h, to allow DMS formation, after which IFN-β treatment was performed for another 24 h. In the latter experiment, the effect of IFN-β treatment on double-membrane sheet formation was markedly decreased: only 19.9% of the DMS-containing cell profiles contained sheets instead of 34.4% in cells treated with IFN-β from the moment of induction of nsp2-3GFP expression. This renders the alternative scenario—IFN-β treatment disrupting existing DMVs and converting them into double-membrane sheets—less likely and strongly suggests that IFN-β treatment indeed influences DMV biogenesis, although we cannot exclude some effects on existing structures. Our data may also shed more light on DMV morphogenesis, since they reinforce the notion that the enwrapping model is a prominent pathway for DMV formation (21, 23). The fact that DMVs were still present after IFN-β treatment suggests that either the enwrapping pathway was only partially blocked or that the remaining DMVs were formed via an alternative mechanism—for example, double budding (23).

We tested the involvement of three selected candidate interferon-induced host factors, PLSCR1, viperin, and CH25H (36–38). When CRISPR/Cas9 knockout cells lacking viperin or PLSCR1 were IFN-β treated, the number of cells showing double-membrane sheets did not decrease. This indicated that although those ISGs were abundantly expressed after IFN-β treatment, neither of them had any impact on DMS formation in our study. CH25H turned out not to be expressed after IFN-β signaling in HuH-7 cells, and treatment of cells with 25HC, the antiviral metabolite synthesized by CH25H, did not visibly affect the EAV nsp2-3-induced DMS. Based on these observations, we concluded that the disruptive effect of IFN-β treatment on DMV formation was unlikely caused by PLSCR1, viperin, or CH25H.

In order to identify the IFN-β-induced factors sought, the best approach would be an unbiased screen of all ISGs expressed after IFN-β treatment. Because of the time-consuming type of EM analysis required to identify DMS, high-throughput EM analysis of all ISGs is not feasible, and screening would have to rely on other materials and methods, such as pull-down assays or colocalization studies that could reveal promising candidates, which could then be analyzed by EM. A previous high-throughput ISG screen, in which the effect of individual ISGs on virus infection was evaluated using fluorescent reporter viruses, included EAV-GFP (39). Most hits in that screen were well-characterized ISGs, such as the OAS proteins, which are known to affect other stages of the viral replication cycle or components in the innate immune signaling cascade, such as IRF1, which lead to the expression of a variety of ISGs. This study did not yield obvious candidates that could be responsible for the observed effects on EAV-induced membrane structures, although some of these could be tested to confirm this. Other possible candidates that we have not explored yet are transmembrane ISGs, such as members of the membrane-anchored IFITM protein family. Because their main mode of action seems to be the blocking of virus entry from endosomes (40), we do not consider these likely responsible for the effects on replication organelles at present.

Interestingly, the type II interferon (gamma interferon gamma [IFN-γ]) pathway has recently been shown to interact with the LC3 conjugating system, which is an established component of the autophagy machinery. LC3 was shown to label the vacuolar membrane of the parasite *Toxoplasma gondii* (41) and also the replication organelles induced by murine norovirus, another positive-strand RNA virus. This serves to recruit immunity-related GTPases induced by IFN-γ signaling to disrupt these membranes and control *T. gondii* and norovirus infections (41, 42). These pathogen-restricting effects did not depend on the degradative functions of autophagy: rather LC3 seems to function as a “flag” that labels foreign membrane structures. Whether similar mechanisms are also in play upon IFN-β treatment has not been investigated, but is an interesting possibility. Since LC3 (although in some cases also in its nonlipidated form) has been found to colocalize with nidovirus replication organelles (43, 44), it would be interesting to investigate whether this could be related to antiviral IFN-β or IFN-γ effects.

The impact of the disruption of DMS formation following IFN-β treatment still requires validation in infected cells, which would be most easily achieved if the responsible factor(s) was identified, because we could then evaluate the effect of this specific factor on replication organelles during infection. If the reduction in DMS prevalence that was observed after IFN-β treatment is representative for what happens during an infection, this could directly reduce overall replication efficiency. It should be noted though, that in the case of coronaviruses, the size and number of DMVs observed during infection was reported not to correlate with viral fitness in cell culture (45, 46). However, impairment of DMV biogenesis could also indirectly affect viral replication, based on their proposed role in the shielding viral PAMPs from cytosolic innate immune sensors. Even though the precise location of arterivirus RNA synthesis remains unclear, the interior of DMVs in EAV-infected cells labels strongly for dsRNA and is enriched for phosphorus, suggesting the abundant presence of viral RNA (13). The decrease of the number of closed DMVs, as observed after IFN-β treatment, could render this material accessible to cytosolic innate immune sensors and promote their recognition as a PAMP (47). Similarly, the exposure of viral RNA to the antiviral action of ISGs could be enhanced: for example, to the ISGs that target viral RNA directly, such as the OAS proteins that indeed very effectively restrict EAV replication if overexpressed (39).

In future research, it would be interesting to investigate whether EAV and other positive-strand RNA viruses counteract innate immune responses targeted at virus-induced membrane structures, as so many other immune responses are counteracted by viral evasion mechanisms. Interestingly, the PLP2 protease in arterivirus nsp2 was shown to reverse posttranslational conjugation of ubiquitin and ISG15 conjugation to cellular targets (47–49). Since both conjugation systems are thought to play a role in innate immune responses, these viral activities could be linked to countering the innate immune-triggered disruption of replication organelles.

In conclusion, we here describe a previously unknown effect of IFN-β treatment on viral replication organelles, which is distinct from effects on the HCV membranous web described earlier. This implies that the innate immune system possesses multiple ways to counteract the formation of replication organelles, thus augmenting the importance of these virus-induced double-membrane structures as a target of the antiviral IFN-β response. HCV and EAV both belong to the DMV-forming class of viruses (4), and it would be very interesting if membrane structures formed by spherule-forming viruses are also targeted by the innate immune system. If so, the next question would be whether the same or different ISGs are involved. Given the rather large body of literature describing the ultrastructure of viral replication organelles, surprisingly little is known about their actual function during virus replication and the reasons why these replication organelles are such a conserved and prominent feature of positive-strand RNA virus replication. Further investigation of the interactions between antiviral innate immunity and replication organelles will likely provide clues on this matter.

## MATERIALS AND METHODS

### Cells, viruses, and antibodies.

HuH-7 cells (Bartenschlager lab, Heidelberg University) were grown in Dulbecco’s modified Eagle’s medium (DMEM; Lonza) supplemented with 8% (vol/vol) fetal calf serum (FCS; Bodinco), 2 mM l-glutamine (PAA Laboratories, Pasching, Austria), and nonessential amino acids (PAA Laboratories). 293T cells (Virgin lab, Washington University School of Medicine in St. Louis) and wild-type C57BL/6 MEFs (Chen lab; University of Texas Southwestern Medical Center) were cultured in DMEM with 10% (vol/vol) FCS. All cell culture media contained 100 U/ml penicillin and 100 µg/ml streptomycin. EAV (Bucyrus strain) and recombinant EAV-GFP were grown on BHK-21 cells as described previously (33). Infections were carried out at a multiplicity of infection (MOI) of 10 unless otherwise indicated and incubated at 37°C. Innate immune stimulation was performed by addition of human interferon-β (IFN-β; PBL) at a concentration of 500 U/ml unless otherwise indicated. 25HC (Sigma) was dissolved in ethanol before use at the indicated concentrations. Cell viability assays were performed using CellTiter 96 AQueous nonradioactive cell proliferation assay (Promega) according to the manufacturer’s instructions. GFP and cell viability assays were analyzed using a Mithras LB940 (Berthold) in 96-well plates.

The primary antibodies used were mouse anti-HA (clone HA.C5; Abcam, Inc.), mouse anti-β-actin (clone AC-74; Sigma), mouse anti-viperin (clone MaP.VIP; Millipore), and goat anti-PLSCR1 (clone N-17; Santa Cruz). The mouse anti-ISG15 antibody was kindly provided by Ernest Borden (Cleveland Clinic). Antibodies recognizing EAV proteins were described previously: rabbit anti-EAV-nsp2 (49) and mouse anti-EAV-N (50).

### Plasmid construction.

Expression constructs that contain sequences that code for EAV nsp2 and nsp3 were assembled in pDONR201 (Life Technologies, Inc.) with an HA tag at the N terminus of nsp2 and enhanced GFP (eGFP) fused to the C terminus of nsp3. The pDONR construct was then transferred using LR Clonase II (Life Technologies, Inc.) to either pcDNA3.1-DEST or pLenti6.3/TO/V5-DEST (Life Technologies, Inc.). Helper plasmids for lentivirus particle production have been described previously (51), and pLenti3.3/TR carrying the *tetR* gene was purchased from Life Technologies, Inc. (34). To make CRISPR/Cas9 knockout cell lines, the LentiCrisprv2 vector was used as previously described (52, 53). Guide RNA sequences are listed in Table S1 in the supplemental material (54).

### Production of lentivirus particles and creation of stable cell lines.

293T cells were transiently transfected with the helper plasmids and either the pLenti3.3/TR vector, the pLenti6.3 expression constructs, or pLentiCrisprv2 using polyethyleneimine (PEI; Sigma). Seventy-two hours after transfection, the supernatant was harvested, spun down for 10 min at 2,000 rpm, filtered through a 0.45-µm-pore filter, and stored at −80°C until use. Subconfluent HuH-7 cells were transduced with pLenti3.3/TR lentiviral particles in the presence of 8 µg/ml Polybrene (Sigma-Aldrich), and after 48 h, cells were passaged, and transduced cells were selected with 100 µg/ml G418 (Life Technologies, Inc.). After several passages, the resulting cell line, named HuH-7/tetR, was transduced again with pLenti6.3 constructs containing the nsp2-3GFP (pLenti6.3/HA-nsp2-3GFP) coding sequence, and cells were selected with 12.5 µg/ml blasticidin S (PAA Laboratories). Expression of transgenes was induced by the addition of 1 µg/ml tetracycline (Life Technologies, Inc.) to the culture media for 24 h. The resulting cell line, HuH-7/tetR/HA-nsp2-3GFP, was transduced with lentivirus particles made with the pLentiCrisprv2 vector with guide RNAs targeting viperin or PLSCR1. All lentivirus-transduced cells were maintained as polyclonal cell pools to avoid clonal differences between control cell lines and knockouts after serial transductions and selections, which could potentially delude the results. Cells were passaged at least 10 times before use in experiments to avoid lingering innate immune responses due to lentiviral transduction.

### Western blotting.

Samples were lysed directly in 2× Laemmli sample buffer (50 mM Tris-HCl [pH 6.8], 20% [vol/vol] glycerol, 4% [wt/vol] sodium dodecyl sulfate [SDS], 20 mM dithiothreitol, 0.02 mg/ml bromophenol blue) and separated on SDS-polyacrylamide gels. Samples were transferred to polyvinylidene difluoride (PVDF) membranes (Amersham) using a Trans-Blot Turbo transfer system (Bio-Rad) and blocked with 5% (wt/vol) Elk milk powder (Campina) in phosphate-buffered saline (PBS) supplemented with 0.05% Tween 20. After incubation with specific antibodies, signal was visualized using ECL Plus Western blotting substrate (Thermo-Fisher).

### Immunofluorescence microscopy.

Cells were grown on coverslips and fixed after 24 h of tetracycline and/or IFN-β treatment, or 12 h postinfection (hpi) with 3% (wt/vol) paraformaldehyde (PFA) in PBS. After permeabilization in 0.2% (vol/vol) Triton X-100, coverslips were incubated with antibodies diluted in 5% (wt/vol) bovine serum albumin (BSA). Nuclei were visualized using 1 µg/ml Hoechst 33258, and samples were embedded using ProLong Gold (Life Technologies, Inc.). Samples were visualized using confocal laser scanning microscopy with a Leica TCS SP8 microscope, which was equipped with a 63× objective (NA, 1.40; 1 Airy unit) and a Leica HyD hybrid detector.

### Flow cytometry.

Cells were harvested using trypsin after 24 h of tetracycline and/or IFN-β treatment and fixed in suspension in 3% (wt/vol) PFA in PBS. Cells were then washed with PBS and stored in PBS with 1% (wt/vol) BSA until analysis. Intracellular GFP levels were measured on a BD FACSCalibur, and data were analyzed using FlowJo software (FlowJo Enterprise).

### Reverse transcriptase quantitative real-time PCR.

RNA was isolated from cells using the NucleoSpin RNA kit (Macherey-Nagel) and converted into cDNA using the RevertAid first-strand cDNA synthesis kit (Thermo) using oligo(dT)_18_ primers. Quantitative real-time PCR (RT-qPCR) was performed using iQ SYBR green SuperMix (Bio-Rad) and a CFX384 real-time PCR detection system (Bio-Rad). Gene-specific primers were used to amplify interferon-stimulated genes as well as genes coding for β-actin and GAPDH (glyceraldehyde-3-phosphate dehydrogenase) as reference genes (Table S1).

### Electron microscopy.

To compare in detail the DMSs induced in the HuH-7/tetR/HA-nsp2-3GFP cell line with those present in EAV-infected HuH-7 cells, high-pressure frozen and freeze-substituted samples of both conditions were prepared and analyzed by electron tomography as described previously (23). All of the samples used for quantifications in this study were fixed in 1.5% (wt/vol) glutaraldehyde in 0.10 M cacodylate buffer (pH 7.4) for 1 h at room temperature. After being washed with 0.14 M cacodylate buffer, samples were postfixed and stained with 1% (wt/vol) osmium tetroxide in 0.10 M cacodylate buffer for 1 h at 4°C. Following subsequent washing with 0.14 M cacodylate buffer and Milli-Q water, cells were scraped and spun down in heated 3% (wt/vol) agar in PBS. After solidification, cell pellets were excised and cut into small blocks, dehydrated in increasing concentrations of ethanol, and embedded in an epoxy resin (LX-112; Ladd Research). After polymerization, 100-nm sections were cut from the blocks and placed on mesh-100 electron microscopy grids, poststained with 7% (wt/vol) uranyl acetate and Reynolds lead citrate, and analyzed using an FEI Tecnai 12 BioTwin equipped with an Eagle cooled slow-scan charge-coupled device (CCD) camera (FEI). Mosaic maps were generated as previously described (35) at 6,800× magnification and binning 2, which corresponded to a pixel size of 3.2 nm.

### Quantification and statistical analysis.

Mosaic maps of several meshes (approximately 170 by 170 µm per mesh) of an EM grid were analyzed for each sample. Cell profiles that were positive for arterivirus-associated DMSs, cell profiles positive for double-membrane sheets, as well as cell profiles that contained a nucleus were annotated and counted using Aperio ImageScope software (Leica). Data from 3 to 4 meshes were combined and compared to those from parallel control samples. Data sets from individual experiments (e.g., control and IFN-β treated) were randomized prior to manual annotation to avoid detection bias. Statistical analysis of individual experiments was performed either using a chi-square test (1 degree of freedom) comparing the ratios in different conditions of cell profiles positive for DMS (DMS^+^) over the total number of cell profiles containing a nucleus or the fraction of cell profiles containing double-membrane sheets out of all DMS^+^ cell profiles. Statistical analysis of replicate experiments was performed with an unpaired Student’s *t* test.

### Data availability.

One mosaic map of each condition used in this study is available at the DANS data repository as an example (http://dx.doi.org/10.17026/dans-zku-4cgy). For the remaining mosaic maps, contact the corresponding author.

## SUPPLEMENTAL MATERIAL

Figure S1  EAV replication is affected by 25HC treatment. HuH-7 cells were infected with EAV-GFP and from 1 h after infection onwards, cells were treated with the indicated concentrations of 25-hydroxy cholesterol (25HC). At 20 hpi, cells were fixed and GFP levels determined. A noninfected control plate was used for a cell viability assay with MTS [3-(4,5-dimethylthiazol-2-yl)-5-(3-carboxymethoxyphenyl)-2-(4-sulfophenyl)-2H-tetrazolium] performed at the time of fixation to check for cytotoxicity of 25HC treatment. 25HC treatment was verified with RT-qPCR of SREBF2. Error bars represent the standard deviation from quadruplicate determinations. Download Figure S1, TIF file, 0.6 MB

Figure S2  CRISPR/Cas9-mediated knockout of viperin and PLSCR1. Protein expression of genes targeted using CRISPR/Cas9 was analyzed using Western blotting. Expression of nsp2-3 was induced in all cells using 1 µg/ml tetracycline for 24 h, and samples were treated with 500 U/ml IFN-β as indicated. Two different guide RNAs targeting both ISGs were used, each leaving very little residual expression in the polyclonal cell pool. The cell pool with the lowest level of residual expression was used for EM analysis. Download Figure S2, TIF file, 1.5 MB

Table S1  Overview of primers and guide RNAs used for RT-qPCR and CRISPR/Cas9. Table S1, XLSX file, 0.01 MB

## References

[1] Miller S, Krijnse-Locker J 2008 Modification of intracellular membrane structures for virus replication. Nat Rev Microbiol 6:363–374. doi:10.1038/nrmicro1890. 18414501 PMC7096853

[2] den Boon JA, Diaz A, Ahlquist P 2010 Cytoplasmic viral replication complexes. Cell Host Microbe 8:77–85. doi:10.1016/j.chom.2010.06.010. 20638644 PMC2921950

[3] Neuman BW, Angelini MM, Buchmeier MJ 2014 Does form meet function in the coronavirus replicative organelle? Trends Microbiol 22:642–647. doi:10.1016/j.tim.2014.06.003. 25037114 PMC7127430

[4] Romero-Brey I, Bartenschlager R 2016 Endoplasmic reticulum: the favorite intracellular niche for viral replication and assembly. Viruses 8:E160. doi:10.3390/v8060160. 27338443 PMC4926180

[5] Diaz A, Ahlquist P 2012 Role of host reticulon proteins in rearranging membranes for positive-strand RNA virus replication. Curr Opin Microbiol 15:519–524. doi:10.1016/j.mib.2012.04.007. 22621853 PMC3670673

[6] Belov GA, van Kuppeveld FJ 2012 (+)RNA viruses rewire cellular pathways to build replication organelles. Curr Opin Virol 2:740–747. doi:10.1016/j.coviro.2012.09.006. 23036609 PMC7102821

[7] Nagy PD, Barajas D, Pogany J 2012 Host factors with regulatory roles in tombusvirus replication. Curr Opin Virol 2:691–698. doi:10.1016/j.coviro.2012.10.004. 23122856

[8] Paul D, Bartenschlager R 2013 Architecture and biogenesis of plus-strand RNA virus replication factories. J Virol 2:32–48. doi:10.5501/wjv.v2.i2.32. PMC3785047 24175228

[9] Kopek BG, Perkins G, Miller DJ, Ellisman MH, Ahlquist P 2007 Three-dimensional analysis of a viral RNA replication complex reveals a virus-induced mini-organelle. PLoS Biol 5:e220. doi:10.1371/journal.pbio.0050220. 17696647 PMC1945040

[10] Schwartz M, Chen J, Janda M, Sullivan M, den Boon J, Ahlquist P 2002 A positive-strand RNA virus replication complex parallels form and function of retrovirus capsids. Mol Cell 9:505–514. doi:10.1016/S1097-2765(02)00474-4. 11931759

[11] Kujala P, Ikäheimonen A, Ehsani N, Vihinen H, Auvinen P, Kääriäinen L 2001 Biogenesis of the Semliki Forest virus RNA replication complex. J Virol 75:3873–3884. doi:10.1128/JVI.75.8.3873-3884.2001. 11264376 PMC114878

[12] Welsch S, Miller S, Romero-Brey I, Merz A, Bleck CK, Walther P, Fuller SD, Antony C, Krijnse-Locker J, Bartenschlager R 2009 Composition and three-dimensional architecture of the dengue virus replication and assembly sites. Cell Host Microbe 5:365–375. doi:10.1016/j.chom.2009.03.007. 19380115 PMC7103389

[13] Knoops K, Bárcena M, Limpens RW, Koster AJ, Mommaas AM, Snijder EJ 2012 Ultrastructural characterization of arterivirus replication structures: reshaping the endoplasmic reticulum to accommodate viral RNA synthesis. J Virol 86:2474–2487. doi:10.1128/JVI.06677-11. 22190716 PMC3302280

[14] Limpens RW, van der Schaar HM, Kumar D, Koster AJ, Snijder EJ, van Kuppeveld FJ, Bárcena M 2011 The transformation of enterovirus replication structures: a three-dimensional study of single- and double-membrane compartments. mBio 2:e00166-11. doi:10.1128/mBio.00166-11. 21972238 PMC3187575

[15] Romero-Brey I, Merz A, Chiramel A, Lee JY, Chlanda P, Haselman U, Santarella-Mellwig R, Habermann A, Hoppe S, Kallis S, Walther P, Antony C, Krijnse-Locker J, Bartenschlager R 2012 Three-dimensional architecture and biogenesis of membrane structures associated with hepatitis C virus replication. PLoS Pathog 8:e1003056. doi:10.1371/journal.ppat.1003056. 23236278 PMC3516559

[16] Belov GA, Nair V, Hansen BT, Hoyt FH, Fischer ER, Ehrenfeld E 2012 Complex dynamic development of poliovirus membranous replication complexes. J Virol 86:302–312. doi:10.1128/JVI.05937-11. 22072780 PMC3255921

[17] Knoops K, Kikkert M, Worm SH, Zevenhoven-Dobbe JC, van der Meer Y, Koster AJ, Mommaas AM, Snijder EJ 2008 SARS-coronavirus replication is supported by a reticulovesicular network of modified endoplasmic reticulum. PLoS Biol 6:e226. doi:10.1371/journal.pbio.0060226. 18798692 PMC2535663

[18] Maier HJ, Hawes PC, Cottam EM, Mantell J, Verkade P, Monaghan P, Wileman T, Britton P 2013 Infectious bronchitis virus generates spherules from zippered endoplasmic reticulum membranes. mBio 4:e00801-13. doi:10.1128/mBio.00801-13. 24149513 PMC3812713

[19] Wieringa R, de Vries AA, van der Meulen J, Godeke GJ, Onderwater JJ, van Tol H, Koerten HK, Mommaas AM, Snijder EJ, Rottier PJ 2004 Structural protein requirements in equine arteritis virus assembly. J Virol 78:13019–13027. doi:10.1128/JVI.78.23.13019-13027.2004. 15542653 PMC524988

[20] Snijder EJ, Kikkert M, Fang Y 2013 Arterivirus molecular biology and pathogenesis. J Gen Virol 94:2141–2163. doi:10.1099/vir.0.056341-0. 23939974

[21] Pedersen KW, van der Meer Y, Roos N, Snijder EJ 1999 Open reading frame 1a-encoded subunits of the arterivirus replicase induce endoplasmic reticulum-derived double-membrane vesicles which carry the viral replication complex. J Virol 73:2016–2026. 9971782 10.1128/jvi.73.3.2016-2026.1999 PMC104444

[22] Snijder EJ, van Tol H, Roos N, Pedersen KW 2001 Non-structural proteins 2 and 3 interact to modify host cell membranes during the formation of the arterivirus replication complex. J Gen Virol 82:985–994. doi:10.1099/0022-1317-82-5-985. 11297673

[23] van der Hoeven B, Oudshoorn D, Koster AJ, Snijder EJ, Kikkert M, Bárcena M 2016 Biogenesis and architecture of arterivirus replication organelles. Virus Res 220:70–90 doi:10.1016/j.virusres.2016.04.001. 27071852 PMC7111217

[24] Gosert R, Kanjanahaluethai A, Egger D, Bienz K, Baker SC 2002 RNA replication of mouse hepatitis virus takes place at double-membrane vesicles. J Virol 76:3697–3708. doi:10.1128/JVI.76.8.3697-3708.2002. 11907209 PMC136101

[25] Romero-Brey I, Bartenschlager R 2014 Membranous replication factories induced by plus-strand RNA viruses. Viruses 6:2826–2857. doi:10.3390/v6072826. 25054883 PMC4113795

[26] Bowie AG, Unterholzner L 2008 Viral evasion and subversion of pattern-recognition receptor signalling. Nat Rev Immunol 8:911–922. doi:10.1038/nri2436. 18989317 PMC7097711

[27] Yoneyama M, Onomoto K, Jogi M, Akaboshi T, Fujita T 2015 Viral RNA detection by RIG-I-like receptors. Curr Opin Immunol 32:48–53. doi:10.1016/j.coi.2014.12.012. 25594890

[28] Gürtler C, Bowie AG 2013 Innate immune detection of microbial nucleic acids. Trends Microbiol 21:413–420. doi:10.1016/j.tim.2013.04.004. 23726320 PMC3735846

[29] Schneider WM, Chevillotte MD, Rice CM 2014 Interferon-stimulated genes: a complex web of host defenses. Annu Rev Immunol 32:513–545. doi:10.1146/annurev-immunol-032713-120231. 24555472 PMC4313732

[30] Anggakusuma, Romero-Brey I, Berger C, Colpitts CC, Boldanova T, Engelmann M, Todt D, Perin PM, Behrendt P, Vondran FW, Xu S, Goffinet C, Schang LM, Heim MH, Bartenschlager R, Pietschmann T, Steinmann E 2015 Interferon-inducible cholesterol-25-hydroxylase restricts hepatitis C virus replication through blockage of membranous web formation. Hepatology 62:702–714. doi:10.1002/hep.27913. 25999047

[31] Helbig KJ, Eyre NS, Yip E, Narayana S, Li K, Fiches G, McCartney EM, Jangra RK, Lemon SM, Beard MR 2011 The antiviral protein viperin inhibits hepatitis C virus replication via interaction with nonstructural protein 5A. Hepatology 54:1506–1517. doi:10.1002/hep.24542. 22045669 PMC3207276

[32] Melén K, Keskinen P, Lehtonen A, Julkunen I 2000 Interferon-induced gene expression and signaling in human hepatoma cell lines. J Hepatol 33:764–772. doi:10.1016/S0168-8278(00)80308-6. 11097485

[33] van den Born E, Posthuma CC, Knoops K, Snijder EJ 2007 An infectious recombinant equine arteritis virus expressing green fluorescent protein from its replicase gene. J Gen Virol 88:1196–1205. doi:10.1099/vir.0.82590-0. 17374763

[34] Yao F, Svensjö T, Winkler T, Lu M, Eriksson C, Eriksson E 1998 Tetracycline repressor, tetR, rather than the tetR-mammalian cell transcription factor fusion derivatives, regulates inducible gene expression in mammalian cells. Hum Gene Ther 9:1939–1950. doi:10.1089/hum.1998.9.13-1939. 9741432

[35] Faas FG, Avramut MC, van den Berg BM, Mommaas AM, Koster AJ, Ravelli RB 2012 Virtual nanoscopy: generation of ultra-large high resolution electron microscopy maps. J Cell Biol 198:457–469. doi:10.1083/jcb.201201140. 22869601 PMC3413355

[36] Helbig KJ, Beard MR 2014 The role of viperin in the innate antiviral response. J Mol Biol 426:1210–1219. doi:10.1016/j.jmb.2013.10.019. 24157441

[37] Sahu SK, Gummadi SN, Manoj N, Aradhyam GK 2007 Phospholipid scramblases: an overview. Arch Biochem Biophys 462:103–114. doi:10.1016/j.abb.2007.04.002. 17481571

[38] Cyster JG, Dang EV, Reboldi A, Yi T 2014 25-Hydroxycholesterols in innate and adaptive immunity. Nat Rev Immunol 14:731–743. doi:10.1038/nri3755. 25324126

[39] Schoggins JW, MacDuff DA, Imanaka N, Gainey MD, Shrestha B, Eitson JL, Mar KB, Richardson RB, Ratushny AV, Litvak V, Dabelic R, Manicassamy B, Aitchison JD, Aderem A, Elliott RM, García-Sastre A, Racaniello V, Snijder EJ, Yokoyama WM, Diamond MS, Virgin HW, Rice CM 2014 Pan-viral specificity of IFN-induced genes reveals new roles for cGAS in innate immunity. Nature 505:691–695. doi:10.1038/nature12862. 24284630 PMC4077721

[40] Diamond MS, Farzan M 2013 The broad-spectrum antiviral functions of IFIT and IFITM proteins. Nat Rev Immunol 13:46–57. doi:10.1038/nri3344. 23237964 PMC3773942

[41] Choi J, Park S, Biering SB, Selleck E, Liu CY, Zhang X, Fujita N, Saitoh T, Akira S, Yoshimori T, Sibley LD, Hwang S, Virgin HW 2014 The parasitophorous vacuole membrane of Toxoplasma gondii is targeted for disruption by ubiquitin-like conjugation systems of autophagy. Immunity 40:924–935. doi:10.1016/j.immuni.2014.05.006. 24931121 PMC4107903

[42] Park S, Choi J, Biering SB, Dominici E, Williams LE, Hwang S 2016 Targeting by AutophaGy proteins (TAG): targeting of IFNG-inducible GTPases to membranes by the LC3 conjugation system of autophagy. Autophagy 12:1153–1167 doi:10.1080/15548627.2016.1178447. 27172324 PMC4990996

[43] Monastyrska I, Ulasli M, Rottier PJ, Guan JL, Reggiori F, de Haan CA 2013 An autophagy-independent role for LC3 in equine arteritis virus replication. Autophagy 9:164–174. doi:10.4161/auto.22743. 23182945 PMC3552881

[44] Reggiori F, Monastyrska I, Verheije MH, Calì T, Ulasli M, Bianchi S, Bernasconi R, de Haan CA, Molinari M 2010 Coronaviruses hijack the LC3-I-positive EDEMosomes, ER-derived vesicles exporting short-lived ERAD regulators, for replication. Cell Host Microbe 7:500–508. doi:10.1016/j.chom.2010.05.013. 20542253 PMC7103375

[45] Al-Mulla HM, Turrell L, Smith NM, Payne L, Baliji S, Züst R, Thiel V, Baker SC, Siddell SG, Neuman BW 2014 Competitive fitness in coronaviruses is not correlated with size or number of double-membrane vesicles under reduced-temperature growth conditions. mBio 5:e01107-13. doi:10.1128/mBio.01107-13. 24692638 PMC3977362

[46] Beachboard DC, Anderson-Daniels JM, Denison MR 2015 Mutations across murine hepatitis virus nsp4 alter virus fitness and membrane modifications. J Virol 89:2080–2089. doi:10.1128/JVI.02776-14. 25473044 PMC4338892

[47] van Kasteren PB, Beugeling C, Ninaber DK, Frias-Staheli N, van Boheemen S, García-Sastre A, Snijder EJ, Kikkert M 2012 Arterivirus and nairovirus ovarian tumor domain-containing deubiquitinases target activated RIG-I to control innate immune signaling. J Virol 86:773–785. doi:10.1128/JVI.06277-11. 22072774 PMC3255818

[48] Frias-Staheli N, Giannakopoulos NV, Kikkert M, Taylor SL, Bridgen A, Paragas J, Richt JA, Rowland RR, Schmaljohn CS, Lenschow DJ, Snijder EJ, García-Sastre A, Virgin HW 2007 Ovarian tumor domain-containing viral proteases evade ubiquitin- and ISG15-dependent innate immune responses. Cell Host Microbe 2:404–416. doi:10.1016/j.chom.2007.09.014. 18078692 PMC2184509

[49] van Kasteren PB, Bailey-Elkin BA, James TW, Ninaber DK, Beugeling C, Khajehpour M, Snijder EJ, Mark BL, Kikkert M 2013 Deubiquitinase function of arterivirus papain-like protease 2 suppresses the innate immune response in infected host cells. Proc Natl Acad Sci U S A 110:E838–E847. doi:10.1073/pnas.1218464110. 23401522 PMC3587229

[50] MacLachlan NJ, Balasuriya UB, Hedges JF, Schweidler TM, McCollum WH, Timoney PJ, Hullinger PJ, Patton JF 1998 Serologic response of horses to the structural proteins of equine arteritis virus. J Vet Diagn Invest 10:229–236. doi:10.1177/104063879801000302. 9683071

[51] Carlotti F, Bazuine M, Kekarainen T, Seppen J, Pognonec P, Maassen JA, Hoeben RC 2004 Lentiviral vectors efficiently transduce quiescent mature 3T3-L1 adipocytes. Mol Ther 9:209–217. doi:10.1016/j.ymthe.2003.11.021. 14759805

[52] Shalem O, Sanjana NE, Hartenian E, Shi X, Scott DA, Mikkelsen TS, Heckl D, Ebert BL, Root DE, Doench JG, Zhang F 2014 Genome-scale CRISPR-Cas9 knockout screening in human cells. Science 343:84–87. doi:10.1126/science.1247005. 24336571 PMC4089965

[53] Sanjana NE, Shalem O, Zhang F 2014 Improved vectors and genome-wide libraries for CRISPR screening. Nat Methods 11:783–784. doi:10.1038/nmeth.3047. 25075903 PMC4486245

[54] Kusano S, Eizuru Y 2012 Human phospholipid scramblase 1 interacts with and regulates transactivation of HTLV-1 Tax. Virology 432:343–352. doi:10.1016/j.virol.2012.06.019. 22789739

