# Supplementary figures and images for: Antiviral Innate Immune Response Interferes with the Formation of Replication-Associated Membrane Structures Induced by a Positive-Strand RNA Virus
Source: mBio. 2016 Dec 6;7(6):e01991-16. doi: 10.1128/mBio.01991-16 (PMC5142621; doi:10.1128/mBio.01991-16)

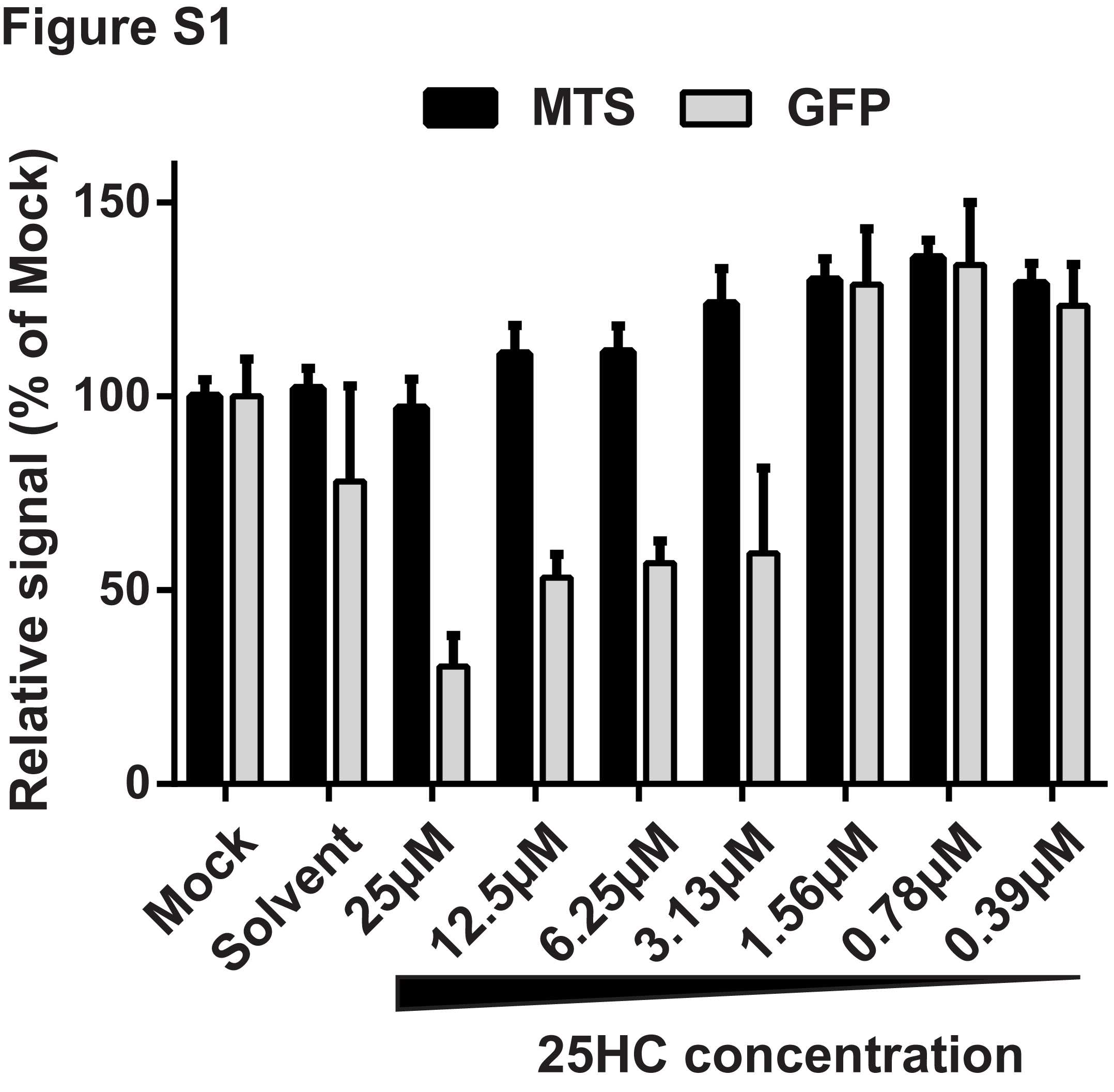

Supplement: Figure S1 — EAV replication is affected by 25HC treatment. HuH-7 cells were infected with EAV-GFP and from 1 h after infection onwards, cells were treated with the indicated concentrations of 25-hydroxy cholesterol (25HC). At 20 hpi, cells were fixed and GFP levels determined. A noninfected control plate was used for a cell viability assay with MTS [3-(4,5-dimethylthiazol-2-yl)-5-(3-carboxymethoxyphenyl)-2-(4-sulfophenyl)-2H-tetrazolium] performed at the time of fixation to check for cytotoxicity of 25HC treatment. 25HC treatment was verified with RT-qPCR of SREBF2. Error bars represent the standard deviation from quadruplicate determinations. Download [file mbo006163092sf1.tif]

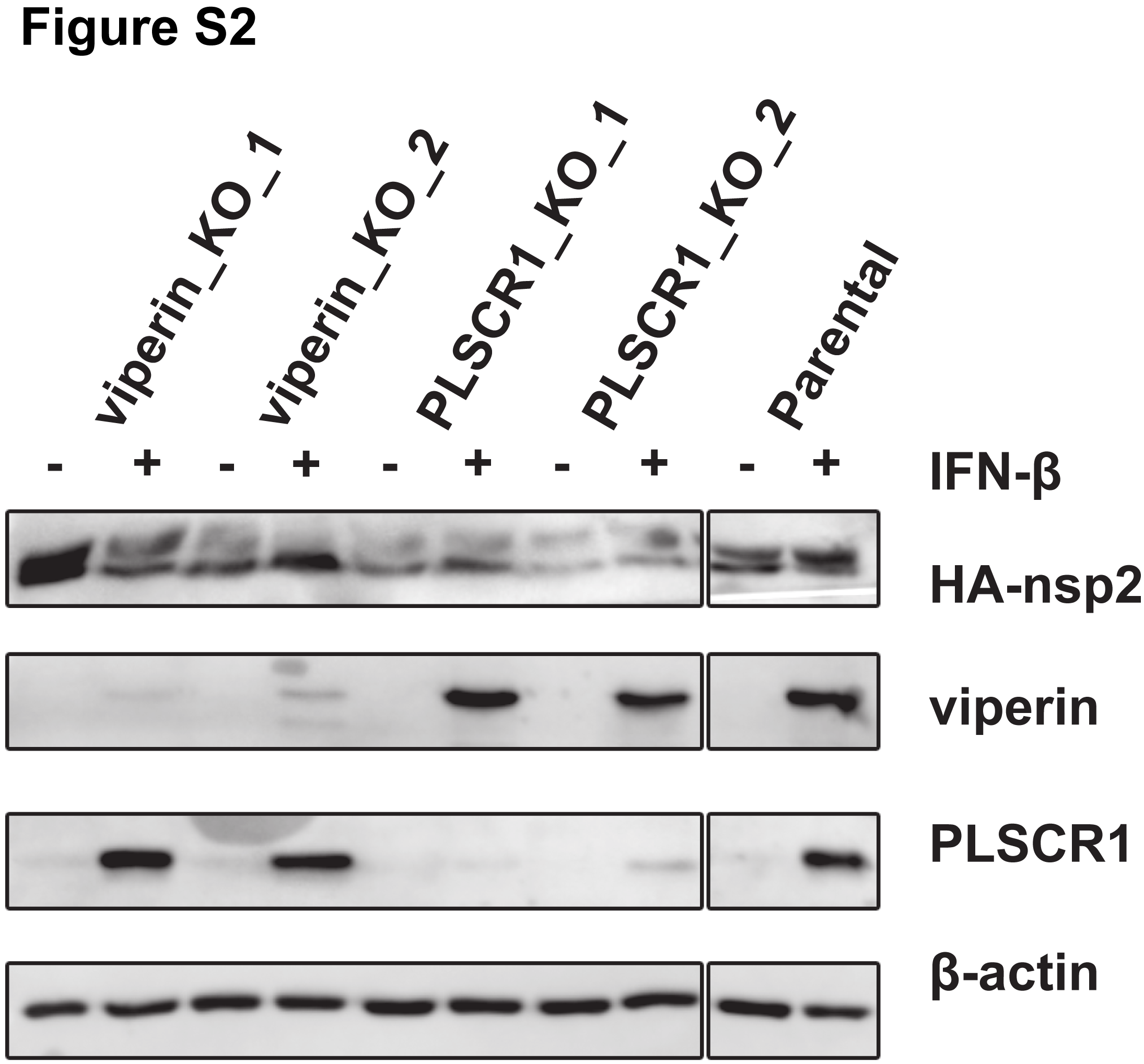

Supplement: Figure S2 — CRISPR/Cas9-mediated knockout of viperin and PLSCR1. Protein expression of genes targeted using CRISPR/Cas9 was analyzed using Western blotting. Expression of nsp2-3 was induced in all cells using 1 µg/ml tetracycline for 24 h, and samples were treated with 500 U/ml IFN-β as indicated. Two different guide RNAs targeting both ISGs were used, each leaving very little residual expression in the polyclonal cell pool. The cell pool with the lowest level of residual expression was used for EM analysis. Download [file mbo006163092sf2.tif]
